# Systematic review of resecting primary tumor in MNETs patients with unresectable liver metastases

**DOI:** 10.18632/oncotarget.14156

**Published:** 2016-12-24

**Authors:** Jingfei Guo, Qian Zhang, Xinyu Bi, Jianguo Zhou, Zhiyu Li, Zhen Huang, Yefan Zhang, Muxing Li, Xiao Chen, Xuhui Hu, Chi Yihebali, Junbo Liang, Jianmei Liu, Jianjun Zhao, Jianqiang Cai, Hong Zhao

**Affiliations:** ^1^ Department of Abdominal Surgical Oncology, Cancer Hospital, Chinese Academy of Medical Sciences and Peking Union Medical College, Beijing, P.R. China; ^2^ Academy of Medical Sciences, Peking Union Medical College, Beijing, China; ^3^ Department of Gastroenterology, Beijing Tiantan Hospital, Capital Medical University, Beijing, China; ^4^ Department of Internal Medicine, Cancer Hospital, Chinese Academy of Medical Sciences and Peking Union Medical College, Beijing, P.R. China; ^5^ State Key Laboratory of Medical Molecular Biology, Institute of Basic Medical Sciences, Chinese Academy of Medical Sciences, Peking Union Medical College, Beijing, China; ^6^ Laboratory of Cell and Molecular Biology, Cancer Hospital, Chinese Academy of Medical Sciences and Peking Union Medical College, Beijing, P.R. China

**Keywords:** midgut neuroendocrine tumor, liver metastasis, palliative surgery, primary tumor resection, survival benefit

## Abstract

**Background:**

Treatment for midgut neuroendocrine tumor patients with unresectable liver metastasis has long been a controversial issue. This system review aims to summarize existing evidence concerning the value of primary tumor resection in this group of patients.

**Results:**

8 cohort studies were identified for qualitative analysis. None of them strictly met with the inclusion criteria and meta-analysis was impossible. There was a tendency towards better overall survival for the primary tumor resected group in all 8 studies, in which 6 demonstrated significant difference. Progression free survival to liver disease was prolonged and less patients died of liver failure in the resected group.

**Methods:**

MEDLINE, EMBASE and CENTRAL were searched until 2016/7/4 for relevant studies, with primary outcome being overall survival, and secondary outcome being progression free survival, cause of death and symptom relief.

**Conclusions:**

Current evidence supports resection of primary tumor for midgut neuroendocrine tumor patients with liver metastases, but randomized controlled trials are required to reach a final conclusion.

## INTRODUCTION

Midgut neuroendocrine tumors (MNETs) are recognized for the ability to secrete serotonin and are associated with the development of carcinoid syndrome (CS). Considered as rare neoplasm traditionally, the incidence of MNETs has gone through a remarkable increase in recent years. They have already surpassed adenocarcinomas as the most common small bowel tumors [1]. MNETs are known for its indolent course and good long-term survival. Research showed that 10-year overall, cause-specific and relative survival are 36%, 80%, 54%, respectively [2]. MNETs are silent at early stage and many patients are detected incidentally at surgery for bowel obstruction and bowel infarction [3]. Considering its insidious nature, stage IV disease is common at initial diagnosis, with liver metastasis being predominant. Other frequent metastatic sites include lymph node and bone [4, 5].

Radical surgery is the only treatment that offers potential cure for patients with resectable metastasis [6]. Cytoreductive surgery is generally carried out when removing 70–90 per cent of the disease is possible [7]. Unfortunately, around 80% of hepatic metastasis is inoperable at the time of presentation [8], and whether primary tumor resection should be conducted in this context is still disputable. In 2014, EAHPBA offered a proposal based on jury votes to support primary tumor removal [9]. The ENETS 2016 guideline also deemed primary tumor resection as necessary even in the presence of liver or lymph node metastasis [10]. None of these recommendations had robust data support, with evidence level being expert opinion.

Previous research on the topic is scarce and highly different in study design, thus no definite conclusion was drawn. We aimed to update the systemic review in 2012 [11] by adding recent evidence and conducting more comprehensive research.

## RESULTS

### Basic characteristics of studies

**Figure 1:**
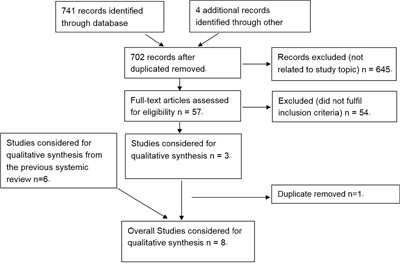
PRISMA diagram showing selection of articles for review

Our literature search identified 748 unique references (Figure 1). After full text review of 57 manuscripts, 3 were selected. Combining these 3 studies with the 6 studies from previous systemic review, we ruled out one duplicate and got 8 distinct studies. Descriptive characteristics of the 8 studies are shown in Table 1 [12–19]. All were retrospective cohort studies, with one multicenter research. Patients from a wide time range from 1960 to 2013 were analyzed. Most studies reported age and/or gender information, while several studies did not mention follow up information. The rest had a median follow up time of 55-90 months.

**Table 1:** Basic characteristics of studies

	Study design	Study duration	Study size	Median Follow up/months	Age	Male %	Intervention compared	Primary Outcome	Secondary Outcome
Givi et al. [12]	Single RCS	1995-2006	84	90	57.9	51.2	Resected versus unresected	OS (MOS, 5-year survival)	Cause of death; PFS of liver disease
Strosberg et al. [13]	Single RCS	1999-2003	146	NR*	60.0	45.5	Resected versus unresected	OS (MOS)	NR
Ahmed et al. [14]	multicenter RCS	1973-2007	360	63.5	61.5	52.5	Resected versus unresected	OS (MOS, 5-year survival)	cause of death
Søreide et al. [15]	Single RCS	1960-1989	75	NR	61	NR	Resected versus unresected	OS (MOS, 5-year survival)	Operation related death
Norlén et al. [16]	Single RCS	1985-2011	603	82.8	63.1	53.9	Resected versus unresected	OS (5-year survival)	Operation related death
van der Horst-Schrivers et al. [17]	Single RCS	1992-2003	76	55	59.4	NR	Resected versus unresected	OS (5-year survival)	NR
S. Pusceddu et al. [18]	Single RCS	1979-2012	139	NR	53	48.2	resected VS unresected	OS (MOS)	NR
Srirajaskanthan, R., et al. [19]	Single RCS	1990-2010	138	NR*	65	49.3%	resected VS unresected	OS(MOS)	NR

* NR=not reported.

** median duration of follow up was 37months and 37.5 months for stage 2 and stage 3 disease, follow up time for stage 1 and stage 4 disease was not reported.

### Quality assessment of studies included

None of the 8 studies strictly met the inclusion criteria (Table 2). In 4 studies [12, 15, 17, 18], MNETs were mixed with NETs from sites other than midgut including pancreas and unknown primary. Only one of the four studies [18] analysed MNETs separately. 7 studies [13–19] included certain proportion of patients without liver metastasis, and only one of them [15] pursued subgroup analysis on hepatic metastatic patients. In regard to intervention, some [13, 14, 17–19] did not differentiate cytoreductive surgery from palliative primary tumor resection. Given the distinct nature of these two types of interventions, we deem it inappropriate to mix them together.

**Table 2:** patient information related to inclusion criteria and reasons to exclude from meta-analysis

Reference	Patients			Intervention
	Patients with midgut -NETS/total^#^	Patients with liver metastasis/total^#^	Liver metastasis strictly defined as unresectable	Primary tumor resection analysed separately from Debulking Surgery
Givi *et al.* [12]	76/84 90.5%	84/84 100%	Yes	Yes
Strosberg *et al.* [13]	146/146 100%	135/146 92%	No	No
Ahmed *et al.* [14]	319/319 100.0%	285/319 89.3%	No	No
Søreide *et al.* [15]	65/75 86.7%	56/75 74%	No	Yes
Norlén *et al.* [16]	603/603 100%	366/601*** 60.9%	No	Yes
van der Horst-Schrivers *et al.* [17]	54/76 71.1%	61/76 80.2%	No	No
S. Pusceddu *et al.* [18]	57/139 41.0%	115/139 82.7%	No	No
Srirajaskanthan, R., *et al.* [19]	138/138 100%	91/138**** 65.9%	No	No

^#^ total refers to patients include for survival analysis.

* 41 patients with unknown primary was included in the study but excluded from the survival analysis concerning resection of primary tumor.

** only 74% of the patients had liver metastasis but subgroup analysis on survival benefit was conducted.

*** data missing for two patients.

**** there were 91 patients with stage 4 disease in the study, patients with liver metastasis were not specifically mentioned.

Results of quality assessment by NOS score are shown in Table 3. 3 studies had a few flaws regarding comparability [13, 15, 18]. None of them analysed baseline difference between primary tumor resected and unresected group, nor did they conduct multivariate analysis to eliminate this difference. 3 studies failed to demonstrate that follow-up period was long enough for outcomes to occur [15, 18, 19]. Above all, all 8 studies scored above 5 and we decided to conduct qualitative analysis on them.

**Table 3:** Quality evaluation with NOS

Reference	Selection	Comparability	Outcome	Total score
Givi *et al.* [12]	4	1	3	8
Strosberg *et al.* [13]	4	0	3	7
Ahmed *et al.* [14]	4	1	3	8
Søreide *et al.* [15]	4	0	2	6
Norlén *et al.* [16]	4	2	3	9
van der Horst-Schrivers *et al.* [17]	4	2	3	9
S. Pusceddu *et al.* [18]	4	0	2	6
Srirajaskanthan, R., *et al.* [19]	4	2	2	8

### Outcomes

#### Primary outcome-overall survival

The 8 studies included a total of 1698 patients in survival analysis (range 65-601), the number of patients underwent primary tumor resection was 1202 (range 27-493). From Table 4, we can see that all of them showed a trend towards better overall survival in the primary resected group, expressed as MOS [12–15, 18, 19] and/or 5-year overall survival rate [12, 14–17]. MOS was 75-141 months and 37-88 months in primary tumor resected and unresected group. 5-year overall survival were 35.7%-81% and 5.4%-46% in these two groups. Two studies demonstrated no statistical difference in OS [13, 17].

**Table 4:** Data on overall survival

Reference	number of patients		Mean Overall Survival		5-year survival rate		Statistically significant or not
	Resected	unresected	Resected	unresected	Resected	unresected	
Givi et al.* [12]	60	24	108 months	50 months	81%	21%	Yes(univariate analysis, no significant difference in patient characteristics & other treatment)
Strosberg *et al.* [13]	100	46	110 months	88 months	NR	NR	No (Univariate Analysis)
Ahmed *et al.* [14]	209	110	119 months	57 months	74%	46%	Yes (multivariate analysis)
Søreide *et al.* [15]	53	12	139 months	69 months	NR	NR	Yes**
Norlén *et al.* [16]	493	86	NR	NR	75%	28%	Yes (multivariate analysis)
van der Horst-Schrivers *et al.* [17]	27	49	75 months	52 months	57%	44%	No (multivariate analysis)
S. Pusceddu *et al.* [18]	92	47	141 months	37 months	NR	NR	Yes(irrespective of histology, other confounders not analysed)
Srirajaskanthan, R., *et al.* [19]	100***	38***	120 months***	56 months***	NR	NR	Yes (multivariate analysis)

* Patients with unsuccessful attempts for primary tumour resection were included in the resected group.

** Unknown statistical analysis method.

*** These were results for all patients. Results for stage IV patients (n=91) was 105 VS 56 months (statistically significant on multivariate analysis).

### Secondary outcomes

None of the studies presented comparison of symptom relief between the two groups, and secondary outcomes were mainly focused on cause of death, including liver metastasis related, primary tumor related as well as operation related death (Table 5). According to Givi *et al*, we can conclude that PFS to liver lesion was prolonged and liver metastasis related death (liver failure) had a significant decrease after removal of the primary tumor [12]. Regarding primary tumor related death, there were conflicting results on bowel obstruction [12, 14], while death caused by bowel infarction obviously reduced after surgery [12]. For surgery induced death, the 30-day post-surgical death rate ranged from 1.43-1.6% [14, 16], and death rate was higher if patients went through a second operation for the midgut lesion [16].

**Table 5:** Data on cause of death

Reference	Cause of death	Primary resected	Primary unresected
Givi *et al.* [12]	Liver failure	75%	82%
	PFS of liver disease	56 months	25 months
	Bowel obstruction	12.5%	0%
	Bowel infarction	0%	12%
	Others & Unknown	12.5%	6%
Ahmed *et al.* [14]	Bowel obstruction related cachexia	4.78%	12.72%
	Died within 30 days of surgery	1.43%	0%
Søreide *et al.* [15]	Post operative mortality rate	2%	0%
Norlén *et al.* [16]	Overall surgery-related 30-day mortality	1.6 %	0%
	30-day mortality after first primary resection	0.5%	0%
	30-day mortality after second primary resection	2.0%	0%

### Subgroup analysis

Different patient characteristics such as presence of carcinoid syndrome, Ki67 status/tumor staging could affect the results. Operation strategies also contribute to heterogeneity of outcome. Relevant information are as follow.

Givi *et al*. [12] analyzed the impact of primary tumor resection on asymptomatic patients. They identified 28 patients without bowel obstruction, bowel infarction and other forms of acute abdomen before operation and compared them to 18 unresected patients. The survival benefit turned out to be statistically significant on univariate analysis.

Norlén *et al*. [16] compared the difference between primary tumor resection alone and along with mesenteric lymphnode resection. 293 patients were radically resected while 200 patients only had primary tumor removed. 5-year survival rate were 77% and 63% for lymphnode resected and unresected group. 10-year overall survival were 52% and 38% respectively. The difference was significant on univariate analysis.

## DISCUSSION

For MNETs patients with unresectable liver metastasis, primary tumor resection is conducted for two reasons, one is to relieve symptoms caused by primary tumor and the other is to improve survival. For symptomatic patients, symptom relief acts as major drive to operate on primary tumor. Decision to resect clinically-silent primary tumors in the presence of unresectable metastasis is particularly hard to make, for possible benefit only lies in extending survival and risk of surgery has to be weighed carefully.

All of the 8 included studies showed a tendency towards better survival for the primary tumor resected group, with 6 of them demonstrated difference that was statistically significant. The problem is to what extent can these results be trusted. We tried to address this issue from two aspects. One is whether primary tumor removal extend survival for real according to the 8 included studies. The other is whether there are evidences to support the underlying reasons favouring primary tumor resection.

There were no RCTs on this issue, making it impossible to draw a definite conclusion. Efforts were made on both design and analysis to correct selection bias and bias caused by loss-of-follow up, but results from retrospective cohort studies should be interpreted with caution.

Another factor leading to uncertainty of result is population deviation from patients we were interested in to patients included in the studies. Some studies included patients other than MNETs, with part of the patients having pancreatic NETs (PNETs) or NETs from unknown primary sites. Several studies included patients with metastasis of sites other than the liver, such as locoregional metastasis, bone metastasis, distant lymphnode metastasis *etc*. However, 7 of the 8 studies focused on advanced-stage NETs with a vast majority of patients having distant metastasis, except for one study [16] in which only 397 of the 603 patients had stage IV disease.

PNETs patients were the main group of people mixing with MNETs in the studies. We found that the role of primary tumor resection in the presence of unresectable liver metastasis in PNETs was also unclear. Several studies did show a survival benefit for primary tumor resection among PNETs patients, and the difference between resected and unresected group was almost as evident as the MNETS [20, 21]. However, weaker recommendation was made for primary tumor resection for PNETs compared with gastrointestinal NETs, arguing that PNETs have worse prognosis and more operation complications [9, 22]. Some studies even showed shorter survival after resecting the primary tumor [23]. What we can conclude is that survival benefit for primary tumor resection in MNETs is no less than that in PNETs. The concern that mixing PNETs with MNETs patients might exaggerate survival benefit can thus be eliminated.

Mixing liver metastasis with other distant metastasis is another problem to address. Since liver is the most common site of distant metastasis in MNETs, it was no surprise to find little data available concerning primary tumor resection in the presence of other distant metastasis. However, if we accept the basic assumption that the worse prognosis a metastasis indicates, the less benefit can be derived from primary tumor resection, we are able to make a rough estimation. Irvin *et al*. found that in MNETs, hazardous ratio of OS for distant metastasis as a whole, liver metastasis, liver involvement > 10% and liver metastasis > 5 were 2.7, 1.79, 2.81 and 3, respectively for univariate analysis. For multivariate analysis, the hazardous ratio for distant metastasis as a whole, liver metastasis and liver involvement > 10% were 1.98, 2.3 and 2.63, respectively [24]. The influence of liver metastasis on prognosis of MNETs is still controversial [25]. According to a study of 256 MNETs patients, individuals with local disease (primary tumor and/or locoregional lymph node metastases) exhibited a median survival of 108 months, compared to 159 months if < 5 liver metastases and 53 months if ≥5 liver metastases [26]. On the other hand, definite conclusion has already been reached that survival for distant metastatic MNETs patients was significantly worse than those without [27–29]. All the above evidence showed that liver metastasis has almost the same, if not better, prognosis compared with other distant metastasis. Therefore, benefit of resecting primary tumour is unlikely to be impaired if patients with other distant metastasis are excluded.

From the above analysis, we can summarize that population deviation caused by confusion with NETs from other primary sites and NETs with other metastasis won't twist the result dramatically. The survival benefit of removing the primary tumor is more likely to be underestimated than overestimated in the studies included.

The next question is whether this survival benefit remains for asymptomatic patients. Givi *et al*. [12] analyzed the impact of primary tumor resection on asymptomatic patients only and found the survival benefit to be statistically significant. However, given the small number of patients involved, we still need more evidences to confirm the results. For operation approach, Norlén *et al*. [16] found that resection of locoregional metastatic lymphnodes was more favourable compared with removing primary tumor alone. According to a research on 1374 MNETs patients of all tumor stages, removal of locoregional metastatic lymph nodes was related with better overall survival on multivariate analysis [30]. More research need to be done to verify this result on patients with hepatic metastasis.

Cause of death for MNETs patients with unresectable liver metastasis includes: primary tumor burden related death, liver metastasis related death, death related to carcinoid syndrome, treatment related death, and death irrelevant to tumor, which is approximately 40-50% due to good long-term survival [31, 32]. Srirajaskanthan, R. *et al*. [19] reported that three main causes of tumor-related death for MNETs were tumor burden(47.7%), small bowel obstruction(13.6%) and carcinoid heart disease (11.4%). Ahmed *et al*. [14] showed the most frequent disease-specific death causes for liver metastatic MNETs to be tumor progression (48%), small bowel obstruction (15%), and carcinoid heart disease (7%).

Tumor burden from liver metastasis is a main death cause for MNETs According to Givi *et al* [12], 79% of all death were due to liver failure during a median follow-up time of 90 months. The study also showed significant decline in death related to liver metastasis (liver failure) after resection of primary tumor and PFS of liver lesion was extended correspondingly from 25 months to 56 months. This was a crucial finding, in that it clarified that resection of primary tumor was advantageous to controlling liver metastasis in MNETs. The impact of primary tumor removal on metastatic disease is controversial in multiple neoplasms including those of the gastrointestinal tract [33, 34]. Some argued that growth rate of liver metastasis would increase due to post-operative immunodeficiency [35, 36], and this adverse effect could also apply to MNETs. Apart from the study of Givi *et al*. [12], no other research was found to address the problem of how primary tumor resection affect liver metastasis. Future studies should investigate into the issue.

Primary tumor burden is another fundamental factor leading to death. Most patients died of bowel obstruction or infarction. From the studies carried out by Givi and Ahmed *et al*. [12, 14] we concluded that there was a slight decrease in death rate directly related to primary tumor after its removal. This slight decrease, however, has to be interpreted under the condition that more patients had symptoms related to primary tumor before operation in the resected group, so it was possible that resection did reduce death caused by primary tumor burden. Interestingly, Givi and Ahmed *et al* [12, 14] showed opposite results on bowel obstruction related death. Contradicting to common expectation, Givi *et al*. [12] reported 2 patients (12.5%) died of bowel obstruction in the resected group and no patients in the unresected group. This could be explained by more advanced pre-operation bowel disease in the resected group, equally possible was that operation could hardly achieve adequate tumor resection and instead led to post-surgical intestinal adhesion.

Another important death-causing factor is carcinoid syndrome. Death caused by carcinoid syndrome is mainly due to carcinoid heart disease (CHD) and patients die of progressive heart failure in most cases. The presence of CHD represented an individual risk factor for death in multivariate analysis for MNETs with a HR of 2.04. If tricuspid regurgitation was present, the HR would rise to 2.52 [24]. Treatment of the tumor itself including somatostatin analogue, hepatic dearterialization, and chemotherapy did not typically result in regression of CHD [37, 38]. Chance of survival was even worse for patients underwent chemotherapy due to possible serotonin release from intestinal enterochromaffin cells [38]. But we did notice in the above 8 studies that resection of primary tumor resulted in better survival in patients with carcinoid syndrome. In the research carried out by S. Pusceddu *et al*. [18], all patients enrolled had liver metastatic NETs with carcinoid syndrome. The MOS was 141 months versus 37 months for primary resected and unresected group. The poor overall survival for patients with carcinoid syndrome was confirmed by Strosberg *et al*. [13], who demonstrated a MOS of 53 months regardless of treatment modalities. Therefore, the MOS of 141 months was indeed a significant improvement in survival, demonstrating primary tumor resection to be an effective anti-tumor therapy for CS patients.

One factor that should not be ignored in assessing palliative treatment is intervention related death. From the above studies, we learned that death directly caused by primary resection surgery was low. According to Ahmed and Norlén et al. [14, 16], 30-day post-operation mortality was no more than 1.6%, and the death rate was even lower as 0.5% for primary resection for the first time. As surgical techniques develop constantly, operation related death should not be a problem of main concern.

All the above evidence tends to support primary tumor resection, but again, we have to emphasize that no definite conclusion can be drawn without RCTs. We feel a pressing need for controlled trials with the following features: 1) inclusion criteria for patients strictly defined as MNETs with unresectable liver metastasis. 2) detailed record of patient information that could affect prognosis, including tumor grade, presence of symptoms, presence of carcinoid heart disease, level of 5-HIAA *etc*. and survival analysis for primary tumor resection among these subgroups. 3) careful tracking down of death cause during follow up and recording PFS to midgut lesion and liver disease respectively after primary tumor resection. 4) information concerning symptom relief and quality of life after primary resection should be gathered as well.

In conclusion, current evidence suggests that there are potential benefits to resect primary tumor in MNETs patients with unresectable liver metastasis, and certain results backup possible reasons behind. But no RCTs have been conducted in this field and all results have to be interpreted with caution. Specific population that may benefit the most from this procedure as well as surgical strategy require more investigation.

## MATERIALS AND METHODS

### Inclusion criteria

Two independent reviewers screened the studies according to specific selection and exclusion criteria. Inclusion/exclusion of contentious studies were made in consultation with a third reviewer.

#### Patients

Patients with MNETs and unresectable liver metastasis were considered for analysis, irrespective of tumour grade, extrahepatic disease and tumor functional status.

#### Intervention

Resection of the primary tumor alone and more extensive resection of the mesentery with metastatic lymph nodes were both included in the analysis.

#### Comparison

Two distinct groups with and without primary tumor resection as well as explicit comparison of outcome between the two groups were required.

#### Outcomes

Primary outcome was overall survival (OS), expressed as the proportion of alive patients 1, 3, 5 or 10 years after the intervention, and/or as median overall survival (MOS). Secondary outcomes were cause of death, symptom relief and progression free survival (PFS). PFS was defined as the interval between intervention and disease progression shown on imaging studies.

#### Types of studies

Studies were included regardless of study type, language, publication status or sample size. We intended to analyze randomized controlled trials (RCTs), quasi-RCTS and non-RCTs, but given the likely paucity of high-quality research on the topic, cohort studies were also considered. Case-control studies, case series, case reports and other observational studies were excluded.

### Search strategy

To update the 2012 system review, we used the same search terms to look for records on MEDLINE in the last five years. Literature retrieval was also conducted in EMBASE and CENTRAL, both of which not included in the 2012 review. All databases were searched until 2016/07/04. The computer search was supplemented with a manual search of the primary studies referenced in all retrieved articles. Oral reports from meetings and correspondence were also explored to minimize publication bias. If certain cohort was used in more than one studies, only the most recent and complete version was included. Full search strategies are displayed in Appendix 1. The methodology was developed from the Preferred Reporting Items for Systematic Reviews and Meta-Analyses (PRISMA) statement.

### Data extraction

Two authors independently extracted relevant data including study design, study duration, study size, median follow-up time and year of publication, types of surgery; sex, age, histology, biochemical markers and carcinoid symptoms; OS, PFS, cause of death (treatment-related mortality etc.) and symptom relief.

### Quality assessment

Study quality was assessed by JADAD score for RCTs and NOS for cohort studies. In the event of disagreements, consensus was achieved in discussion with the corresponding author.

### Statistical analysis

Meta-analysis of RCTs or cohort studies was planned using Revman 5.3 with the following methods: calculation of the relative risk with 95 per cent confidence interval for dichotomous variables, calculation of the mean difference for continuous variables, use of a random effects model, evaluation of heterogeneity by χ^2^ test, and measure of the quantity of heterogeneity by means of the I^2^ value.

## SUPPLEMENTARY APPENDIX



## References

[1] Bilimoria KY, Bentrem DJ, Wayne JD, Ko CY, Bennett CL, Talamonti MS (2009). Small bowel cancer in the United States: changes in epidemiology, treatment, and survival over the last 20 years. Annals of surgery.

[2] Zar N, Garmo H, Holmberg L, Rastad J, Hellman P (2004). Long-term survival of patients with small intestinal carcinoid tumors. World journal of surgery.

[3] Modlin IM, Kidd M, Latich I, Zikusoka MN, Shapiro MD (2005). Current status of gastrointestinal carcinoids. Gastroenterology.

[4] Van Loon K, Zhang L, Keiser J, Carrasco C, Glass K, Ramirez MT, Bobiak S, Nakakura EK, Venook AP, Shah MH, Bergsland EK (2015). Bone metastases and skeletal-related events from neuroendocrine tumors. Endocr Connect.

[5] Kos-Kudla B, O’Toole D, Falconi M, Gross D, Kloppel G, Sundin A, Ramage J, Oberg K, Wiedenmann B, Komminoth P, Van Custem E, Mallath M, Papotti M, Caplin M (2010). ENETS consensus guidelines for the management of bone and lung metastases from neuroendocrine tumors. Neuroendocrinology.

[6] Pavel M, Baudin E, Couvelard A, Krenning E, Oberg K, Steinmuller T, Anlauf M, Wiedenmann B, Salazar R (2012). ENETS Consensus Guidelines for the management of patients with liver and other distant metastases from neuroendocrine neoplasms of foregut, midgut, hindgut, and unknown primary. Neuroendocrinology.

[7] Hodul P, Malafa M, Choi J, Kvols L (2006). The role of cytoreductive hepatic surgery as an adjunct to the management of metastatic neuroendocrine carcinomas. Cancer Control.

[8] Yankol Y, Mecit N, Kanmaz T, Acarli K, Kalayoglu M (2015). Living donor liver transplantation: a life-saving option in emergency situations for diffuse hepatic neuroendocrine tumor metastasis. Transplantation proceedings.

[9] Frilling A, Modlin IM, Kidd M, Russell C, Breitenstein S, Salem R, Kwekkeboom D, Lau WY, Klersy C, Vilgrain V, Davidson B, Siegler M, Caplin M, Solcia E, Schilsky R (2014). Recommendations for management of patients with neuroendocrine liver metastases. Lancet Oncol.

[10] Pavel M, O’Toole D, Costa F, Capdevila J, Gross D, Kianmanesh R, Krenning E, Knigge U, Salazar R, Pape UF, Oberg K (2016). ENETS Consensus Guidelines Update for the Management of Distant Metastatic Disease of Intestinal, Pancreatic, Bronchial Neuroendocrine Neoplasms (NEN) and NEN of Unknown Primary Site. Neuroendocrinology.

[11] Capurso G, Rinzivillo M, Bettini R, Boninsegna L, G Delle Fave, Falconi M (2012). Systematic review of resection of primary midgut carcinoid tumour in patients with unresectable liver metastases. The British journal of surgery.

[12] Givi B, Pommier SJ, Thompson AK, Diggs BS, Pommier RF (2006). Operative resection of primary carcinoid neoplasms in patients with liver metastases yields significantly better survival. Surgery.

[13] Strosberg J, Gardner N, Kvols L (2009). Survival and prognostic factor analysis of 146 metastatic neuroendocrine tumors of the mid-gut. Neuroendocrinology.

[14] Ahmed A, Turner G, King B, Jones L, Culliford D, McCance D, Ardill J, Johnston BT, Poston G, Rees M, Buxton-Thomas M, Caplin M, Ramage JK (2009). Midgut neuroendocrine tumours with liver metastases: results of the UKINETS study. Endocrine-related cancer.

[15] Soreide O, Berstad T, Bakka A, Schrumpf E, Hanssen LE, Engh V, Bergan A, Flatmark A (1992). Surgical treatment as a principle in patients with advanced abdominal carcinoid tumors. Surgery.

[16] Norlen O, Stalberg P, Oberg K, Eriksson J, Hedberg J, Hessman O, Janson ET, Hellman P, Akerstrom G (2012). Long-term results of surgery for small intestinal neuroendocrine tumors at a tertiary referral center. World journal of surgery.

[17] van der Horst-Schrivers AN, Post WJ, Kema IP, Links TP, Willemse PH, Wymenga AN, de Vries EG (2007). Persistent low urinary excretion of 5-HIAA is a marker for favourable survival during follow-up in patients with disseminated midgut carcinoid tumours. European journal of cancer (Oxford, England : 1990).

[18] Pusceddu S, Buzzoni R, Mazzaferro V, Pacifici M, Procopio G, Damato A, D’Autilia E, Bertarelli G, Seregni E, De Braud F (2013). Primary tumour resection impact on survival in patient with carcinoid syndrome (CS). Analysis of 139 patients (pts) with well differentiated neuroendocrine tumors (WDNETs) from database of Istituto Nazionale Tumori Milano. European Journal of Cancer.

[19] Srirajaskanthan R, Ahmed A, Prachialias A, Srinivasan P, Heaton N, Jervis N, Quaglia A, Vivian G, Ramage JK (2013). ENETS TNM Staging Predicts Prognosis in Small Bowel Neuroendocrine Tumours. ISRN Oncology.

[20] Bertani E, Fazio N, Botteri E, Chiappa A, Falconi M, Grana C, Bodei L, Papis D, Spada F, Bazolli B, Andreoni B (2014). Resection of the primary pancreatic neuroendocrine tumor in patients with unresectable liver metastases: possible indications for a multimodal approach. Surgery.

[21] Haugvik SP, Janson ET, Osterlund P, Langer SW, Falk RS, Labori KJ, Vestermark LW, Gronbaek H, Gladhaug IP, Sorbye H (2016). Surgical Treatment as a Principle for Patients with High-Grade Pancreatic Neuroendocrine Carcinoma: A Nordic Multicenter Comparative Study. Annals of surgical oncology.

[22] Capurso G, Bettini R, Rinzivillo M, Boninsegna L, G Delle Fave, Falconi M (2011). Role of resection of the primary pancreatic neuroendocrine tumour only in patients with unresectable metastatic liver disease: a systematic review. Neuroendocrinology.

[23] Bettini R, Mantovani W, Boninsegna L, Crippa S, Capelli P, Bassi C, Scarpa A, Pederzoli P, Falconi M (2009). Primary tumour resection in metastatic nonfunctioning pancreatic endocrine carcinomas. Digestive and liver disease.

[24] Modlin IM, Gustafsson BI, Pavel M, Svejda B, Lawrence B, Kidd M (2010). A nomogram to assess small-intestinal neuroendocrine tumor (‘carcinoid’) survival. Neuroendocrinology.

[25] Van Gompel JJ, Sippel RS, Warner TF, Chen H (2004). Gastrointestinal carcinoid tumors: factors that predict outcome. World journal of surgery.

[26] Janson ET, Holmberg L, Stridsberg M, Eriksson B, Theodorsson E, Wilander E, Oberg K (1997). Carcinoid tumors: analysis of prognostic factors and survival in 301 patients from a referral center. Annals of oncology.

[27] Wang Z, Li W, Chen T, Yang J, Luo L, Zhang L, Sun B, Liang R (2015). Retrospective analysis of the clinicopathological characteristics of gastrointestinal neuroendocrine neoplasms. Exp Ther Med.

[28] Caprotti R, Angelini C, Mussi C, Romano F, Sartori P, Scaini A, Muselli P, Uggeri F (2003). Gastrointestinal carcinoids. Prognosis and survival. Minerva Chir.

[29] Srirajaskanthan R, Ahmed A, Ramage JK (2012). Does the TNM staging criteria predict survival in patients with small bowel neuroendocrine tumours?. Gut.

[30] Landry CS, Lin HY, Phan A, Charnsangavej C, Abdalla EK, Aloia T, J Nicolas Vauthey, Katz MH, Yao JC, Fleming JB (2013). Resection of at-risk mesenteric lymph nodes is associated with improved survival in patients with small bowel neuroendocrine tumors. World journal of surgery.

[31] Soreide JA, van Heerden JA, Thompson GB, Schleck C, Ilstrup DM, Churchward M (2000). Gastrointestinal carcinoid tumors: long-term prognosis for surgically treated patients. World journal of surgery.

[32] Landerholm K, Zar N, Andersson RE, Falkmer SE, Jarhult J (2011). Survival and prognostic factors in patients with small bowel carcinoid tumour. The British journal of surgery.

[33] Gustavsson B (2012). Simultaneous surgery for primary colorectal cancer and metastatic lesions?. Scandinavian journal of gastroenterology.

[34] Turanli S (2010). The value of resection of primary tumor in gastric cancer patients with liver metastasis. Indian J Surg.

[35] Slesser AA, Bhangu A, Brown G, Mudan S, Tekkis PP (2013). The management of rectal cancer with synchronous liver metastases: a modern surgical dilemma. Tech Coloproctol.

[36] Gonzalez HD, Figueras J (2007). Practical questions in liver metastases of colorectal cancer: general principles of treatment. HPB.

[37] Denney WD, Kemp WE, Anthony LB, Oates JA, Byrd BF (1998). Echocardiographic and biochemical evaluation of the development and progression of carcinoid heart disease. J Am Coll Cardiol.

[38] Moller JE, Connolly HM, Rubin J, Seward JB, Modesto K, Pellikka PA (2003). Factors associated with progression of carcinoid heart disease. The New England journal of medicine.

