# Supplementary material for: Systematic review of resecting primary tumor in MNETs patients with unresectable liver metastases
Source: Oncotarget. 2016 Dec 24;8(10):17396–405. doi: 10.18632/oncotarget.14156 (PMC5370049; doi:10.18632/oncotarget.14156)
Supplement: Supplementary file 1 [file oncotarget-08-17396-s001.pdf]

Systematic review of resecting primary tumor in MNETs patients with unresectable liver metastases

APPENDIX 1

1. Search strategy from the previous review-records on MEDLINE before 2012

(neuroendocrine tumor OR neuroendocrine tumors OR neuroendocrine tumour OR neuroendocrine tumours OR carcinoid tumor OR carcinoids OR tumors, carcinoid OR carcinoid OR gastrointestinal endocrine carcinoma OR carcinoid syndrome OR gastrointestinal endocrine tumors OR argentaffinoma OR argentaffinomas OR goblet cell carcinoid OR goblet cell carcinoids OR apudoma OR apudomas OR carcinoids, goblet cell OR carcinoid, goblet cell OR carcinoid syndrome, malignant OR carcinoid syndromes, malignant OR malignant carcinoid syndromes OR syndrome, malignant carcinoid OR syndromes, malignant carcinoid OR carcinoid heart diseases OR heart disease, carcinoid OR heart diseases, carcinoid) AND (operative surgical procedure OR operative surgical procedures OR procedures, operative surgical OR surgical procedure, operative OR operative procedures OR operative procedure OR procedure, operative OR procedures, operative OR procedure, operative surgical OR primary resection OR small intestine resection OR ileocecal resection OR right-sided hemicolectomy OR mesenteric dissection OR primary tumour resection) AND (metastatic OR metastasis OR secondary OR spread OR advanced OR metastases, neoplasm OR neoplasm metastases OR metastasis OR metastases OR metastasis, neoplasm OR residual neoplasm OR neoplasms, residual OR residual neoplasms OR residual cancer OR cancer, residual OR cancers, residual OR residual cancers OR residual tumor OR residual tumors OR tumor, residual OR tumors, residual) AND (liver OR hepatic).

2. Search strategy for newly published articles from MEDLINE (2011-2016) and records from EMBASE and CENTRAL

MEDLINE

1#[Patient-Neuroendocrine Tumor]	(neuroendocrine tumors [MeSH Terms]) OR (neuroendocrine tumor OR neuroendocrine tumors OR neuroendocrine tumour OR neuroendocrine tumours OR carcinoid tumor OR carcinoids OR tumors, carcinoid OR carcinoid OR gastrointestinal endocrine carcinoma OR carcinoid syndrome OR gastrointestinal endocrine tumors OR argentaffinoma OR argentaffinoma OR goblet cell carcinoid OR goblet cell carcinoids OR apudoma OR apudomas OR carcinoids, goblet cell OR carcinoid, goblet cell OR carcinoid syndrome, malignant OR carcinoid syndromes, malignant OR malignant carcinoid syndromes OR syndrome, malignant carcinoid OR syndromes, malignant carcinoid)	156886
2# [Patient-Liver Metastasis]	(Neoplasm Metastasis [MeSH Terms]) OR ((metastatic OR metastasis OR secondary OR spread OR advanced OR metastases, neoplasm OR neoplasm metastases OR metastasis OR metastases OR metastasis, neoplasm OR residual neoplasm OR neoplasms, residual OR residual neoplasms OR residual cancer OR cancer, residual OR cancers, residual OR residual cancers OR residual tumor OR residual tumors OR tumor, residual OR tumors, residual)) ***AND*** (liver OR hepatic)	109770
3#[Intervention-Surgery]	(Surgical Procedures, Operative [MeSH Terms]) OR ((operative surgical procedure OR operative surgical procedures OR procedures, operative surgical OR surgical procedure, operative OR operative procedures OR operative procedure OR procedure, operative OR procedures, operative OR procedure, operative surgical OR primary resection OR small intestine resection OR ileocecal resection OR right-sided hemicolectomy OR mesenteric dissection OR primary tumour resection))	3914734
4#[Study type-RCT,quasi-RCT,non-RCT,cohort study]	((((((((((randomized controlled trial [Publication Type]) OR controlled clinical trial [Publication Type]) OR randomized [Title/Abstract]) OR placebo [Title/Abstract]) OR randomly [Title/Abstract]) OR trial [Title/Abstract]) OR groups [Title/Abstract]) OR drug therapy [MeSH Subheading]) AND human [MeSH Terms])) OR cohort	3211776
5# [Final Result]	1# AND 2# AND 3# AND 4# published in the last 5 years	186

CENTRAL

1#[Patient-Neuroendocrine Tumor]	(neuroendocrine tumor OR neuroendocrine tumors OR neuroendocrine tumour OR neuroendocrine tumours OR carcinoid tumor OR carcinoids OR tumors, carcinoid OR carcinoid OR gastrointestinal endocrine carcinoma OR carcinoid syndrome OR gastrointestinal endocrine tumors OR argentaffinoma OR argentaffinoma OR goblet cell carcinoid OR goblet cell carcinoids OR apudoma OR apudomas OR carcinoids, goblet cell OR carcinoid, goblet cell OR carcinoid syndrome, malignant OR carcinoid syndromes, malignant OR malignant carcinoid syndromes OR syndrome, malignant carcinoid OR syndromes, malignant carcinoid)	697
2# [Patient-Liver Metastasis]	((metastatic OR metastasis OR secondary OR spread OR advanced OR metastases, neoplasm OR neoplasm metastases OR metastasis OR metastases OR metastasis, neoplasm OR residual neoplasm OR neoplasms, residual OR residual neoplasms OR residual cancer OR cancer, residual OR cancers, residual OR residual cancers OR residual tumor OR residual tumors OR tumor, residual OR tumors, residual)) ***AND*** (liver OR hepatic)	8933
3#[Intervention-Surgery]	(operative surgical procedure OR operative surgical procedures OR procedures, operative surgical OR surgical procedure, operative OR operative procedures OR operative procedure OR procedure, operative OR procedures, operative OR procedure, operative surgical OR primary resection OR small intestine resection OR ileocecal resection OR right-sided hemicolectomy OR mesenteric dissection OR primary tumour resection)	12684
4# [Final Result]	{and 1#-3#}	63

EMBASE

1#[Patient-Neuroendocrine Tumor]	'neuroendocrine tumor'/exp OR 'neuroendocrine tumor' OR 'neuroendocrine tumors'/exp OR 'neuroendocrine tumors' OR 'neuroendocrine tumour'/exp OR 'neuroendocrine tumour' OR 'neuroendocrine tumours'/exp OR 'neuroendocrine tumours' OR 'carcinoid tumor'/exp OR 'carcinoid tumor' OR 'carcinoids' OR 'tumors, carcinoid' OR 'carcinoid'/exp OR 'carcinoid' OR 'gastrointestinal endocrine carcinoma' OR 'carcinoid syndrome'/exp OR 'carcinoid syndrome' OR 'gastrointestinal endocrine tumors' OR 'argentaffinoma' OR 'goblet cell carcinoid' OR 'goblet cell carcinoids' OR 'apudoma'/exp OR 'apudoma' OR 'apudomas' OR 'carcinoids, goblet cell' OR 'carcinoid, goblet cell' OR 'carcinoid syndrome, malignant' OR 'carcinoid syndromes, malignant' OR 'malignant carcinoid syndromes' OR 'syndrome, malignant carcinoid' OR 'syndromes, malignant carcinoid'	82841
2# [Patient-Liver Metastasis]	'metastatic' OR 'secondary' OR 'spread' OR 'advanced' OR 'metastases, neoplasm' OR 'neoplasm metastases' OR 'metastasis'/exp OR 'metastasis' OR 'metastases'/exp OR 'metastases' OR 'metastasis, neoplasm' OR 'residual neoplasm' OR 'neoplasms, residual' OR 'residual neoplasms' OR 'residual cancer' OR 'cancer, residual' OR 'cancers, residual' OR 'residual cancers' OR 'residual tumor' OR 'residual tumors' OR 'tumor, residual' OR 'tumors, residual' AND ('liver'/exp OR 'liver' OR 'hepatic')	180166
3#[Intervention-Surgery]	'operative surgical procedure'/exp OR 'operative surgical procedure' OR 'operative surgical procedures' OR 'procedures, operative surgical' OR 'surgical procedure, operative' OR 'operative procedures' OR 'operative procedure' OR 'procedure, operative' OR 'procedures, operative' OR 'procedure, operative surgical' OR 'primary resection' OR 'small intestine resection'/exp OR 'small intestine resection' OR 'ileocecal resection' OR 'right-sided hemicolectomy' OR 'mesenteric dissection' OR 'primary tumour resection'	3980252
4#[Study type-RCT,quasi-RCT,non-RCT,cohort study]	'trial' OR 'cohort'	2071765
5# [Final Result]	1# AND 2# AND 3# AND 4#	496
